# Homocysteine Metabolism in Pregnancy and Developmental Impacts

**DOI:** 10.3389/fcell.2022.802285

**Published:** 2022-06-30

**Authors:** Stephen W. D’Souza, Jocelyn D. Glazier

**Affiliations:** ^1^ Maternal and Fetal Health Research Centre, School of Medical Sciences, Faculty of Biology, Medicine and Health, University of Manchester, St Mary’s Hospital, Manchester Academic Health Science Centre, Manchester, United Kingdom; ^2^ Division of Evolution, Infection and Genomics, Faculty of Biology, Medicine and Health, School of Biological Sciences, Manchester Academic Health Science Centre, University of Manchester, Manchester, United Kingdom

**Keywords:** embryo, fetus, folate, methylation, neural tube defects, placenta, vitamin B_12_, yolk sac

## Abstract

Homocysteine is a metabolite generated by methionine cycle metabolism, comprising the demethylated derivative of methionine. Homocysteine can be metabolised by the transsulphuration pathway to cystathionine, which requires vitamin B_6_, or can undergo remethylation to methionine. Homocysteine remethylation to methionine is catalysed by methionine synthase activity which requires vitamin B_12_, regenerating methionine to allow synthesis of the universal methyl donor *S*-adenosylmethionine required for methylation and gene transcription regulation. The methyl-group donated for homocysteine remethylation comes from 5-methyltetrahydrofolate generated by the folate cycle, which allows tetrahydrofolate to be returned to the active folate pool for nucleotide biosynthesis. Therefore the integrated actions of the methionine and folate cycles, required to metabolise homocysteine, also perpetuate methylation and nucleotide synthesis, vitally important to support embryonic growth, proliferation and development. Dysregulated activities of these two interdependent metabolic cycles, arising from maternal suboptimal intake of nutrient co-factors such as folate and vitamin B_12_ or gene polymorphisms resulting in reduced enzymatic activity, leads to inefficient homocysteine metabolic conversion causing elevated concentrations, known as hyperhomocysteinemia. This condition is associated with multiple adverse pregnancy outcomes including neural tube defects (NTDs). Raised homocysteine is damaging to cellular function, binding to proteins thereby impairing their function, with perturbed homocysteine metabolism impacting negatively on embryonic development. This review discusses the “cross-talk” of maternal-fetal homocysteine interrelationships, describes the placental transport of homocysteine, homocysteine impacts on pregnancy outcomes, homocysteine and methylation effects linking to NTD risk and proposes a putative pathway for embryonic provision of folate and vitamin B_12_, homocysteine-modulating nutrients that ameliorate NTD risk.

## Introduction

**FIGURE 1:**
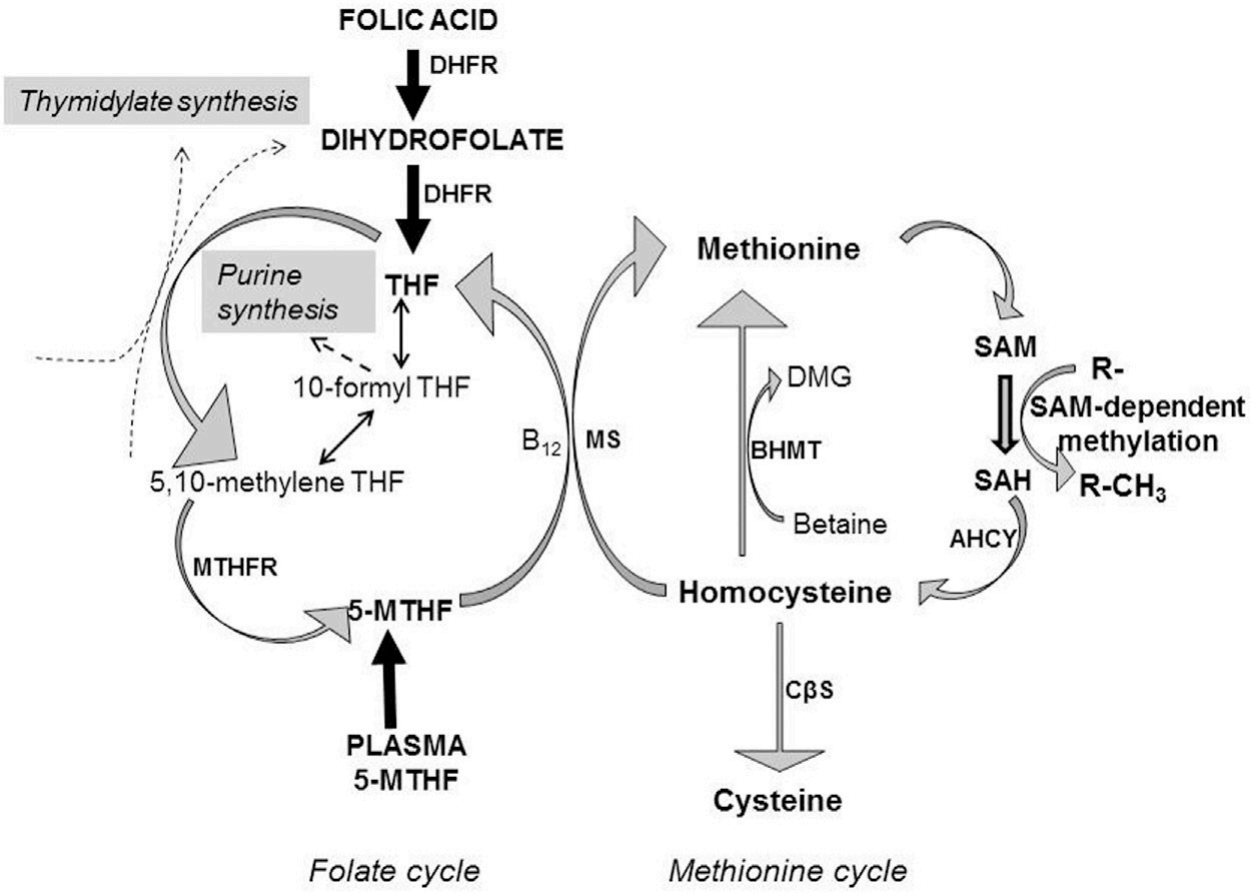
Homocysteine metabolism is dependent on folate and methionine metabolism. Folic acid is reduced to dihydrofolate by the action of dihydrofolate reductase (DHFR), which together with dietary dihydrofolate is reduced further to tetrahydrofolate (THF), which enters the folate metabolic cycle. Alternatively, folate may enter the folate cycle as 5-methyltetrahydrofolate (5-MTHF), with folate cycle metabolism involved in the transfer of one-carbon units to generate thymidylate and purines for nucleotide biosynthesis. Methionine acts as the precursor for the ATP-dependent synthesis of the primary methyl donor *S*-adenosylmethionine (SAM) which through the action of methyltransferases transfer methyl groups to various acceptors (R-) resulting in the methylation of DNA, proteins and lipids, whilst generating *S-*adenosylhomocysteine (SAH). SAH is catabolized to homocysteine by *S*-adenosylhomocysteine hydrolase (AHCY). Homocysteine can be metabolised by three metabolic pathways. 1) Remethylation of homocysteine to methionine relies on 5-MTHF as methyl donor, catalysed by the vitamin B_12_-dependent action of methionine synthase (MS). THF is then re-cycled to form 5-MTHF, catalysed by methylenetetrahydrofolate reductase (MTHFR). 2) Betaine can be utilized as an alternative methyl donor to generate methionine and dimethylglycine (DMG), catalysed by betaine-homocysteine methyltransferase (BHMT). 3) Alternatively, homocysteine can enter the transsulphuration pathway being converted to cysteine through the action of cystathionine β-synthase (CβS).

An optimal maternal dietary intake during pregnancy is essential to provide the balance of nutrients required for normal embryogenesis and fetal growth and development (Williamson, 2016). A constellation of integrative maternal metabolic networks provides nutrient-derived precursors to the developing fetus, which can then be utilised for cellular biosynthesis, bioenergetic regulation and protein accretion, processes all highly dependent on appropriately balanced maternal dietary intakes. Metabolites can act as cell signalling molecules (Finkelstein, 2007), regulating processes such as cellular proliferation (Miyazawa and Aulehla, 2018), leading to an emergent appreciation that metabolites can play a key role in “maternal-fetal crosstalk” during embryonic and fetal development. This has prompted continued interest in elucidating more fully the links between maternal environmental nutrient cues and metabolism and how this affects developmental plasticity and phenotypic outcomes. To this end, there has been considerable interest in the co-ordinated interconnectivity of carbon-directed metabolic pathways, as these are crucial in providing carbon-containing moieties for normal development (Miyazawa and Aulehla, 2018). Within this integrative metabolic network, one-carbon metabolism plays an essential role, encompassing a range of metabolic reactions that interlink the folate and methionine metabolic cycles (Figure 1). Importantly, the interconnected activities of these two cycles allow the transfer of methyl (-CH_3_) groups, while also providing one-carbon units for multiple biosynthetic processes including purine and thymidine synthesis required for nucleic acid synthesis and cellular proliferation (Ducker and Rabinowitz, 2017; Figure 1). Additionally, the integrated activities of these two metabolic cycles allow the synthesis of *S*-adenosylmethionine (SAM; Figure 1), the sole methyl donor for multiple methylation reactions including gene methylation (Bottiglieri, 2002). Methyl donation from SAM to various cellular acceptors generates *S*-adenosylhomocysteine (SAH; Figure 1). The efficient catabolism of SAH (Figure 1) by SAH hydrolase to homocysteine and adenosine is essential as SAH can inhibit the activities of SAM-dependent methyltransferases that participate in a variety of methylation reactions (Finkelstein, 2007).

In this short review, we discuss aspects that relate to maternal-fetal homocysteine “cross-talk” and impacts on pregnancy and embryonic/fetal development, focussing on our previous studies. We present an overview of some key characteristics of homocysteine and its effects in pregnancy, describe maternal-fetal homocysteine relationships in human pregnancy, elaborate on homocysteine transport mechanisms across the placenta, outline the potential protein-modifying cellular effects of homocysteine, make reference to the nutrient-modulation of homocysteine with emphasis on folate and vitamin B_12_, discuss the potential effects of homocysteine with regard to methylation in the aetiology of neural tube defects (NTDs) and propose a pathway for the embryonic provision of folate and vitamin B_12_, nutrient factors that ameliorate NTD risk.

### Homocysteine–an Intermediate Metabolite of the Methionine Cycle That is Associated With Adverse Pregnancy Outcomes

Homocysteine is a thiol group-containing amino acid generated by methionine cycle metabolism that is the demethylated derivative of methionine (Selhub, 1999; Strain et al., 2004; Figure 1). As a branch point metabolite, homocysteine can be converted *via* three potential metabolic pathways, as illustrated in Figure 1: 1) remethylation to methionine using 5-methyltetrahydrofolate (5-MTHF) as the methyl donor catalysed by the action of methionine synthase requiring vitamin B_12_ (cobalamin) as a cofactor, 2) remethylation by betaine as methyl donor to generate methionine and dimethylglycine catalysed by betaine-homocysteine methyltransferase (BHMT) or 3) transsulphuration to cysteine catalysed by cystathionine β-synthase (CβS) requiring vitamin B_6_ (pyridoxine) as a cofactor. 5-MTHF for homocysteine remethylation is generated by the activity of methylenetetrahydrofolate reductase (MTHFR) which requires vitamin B_2_ (riboflavin) to produce flavin adenine dinucleotide as a cofactor for MTHFR enzyme activity. Hence, homocysteine is placed at the intersection of the transmethylation and transsulphuration conversion pathways that require involvement of B-group vitamins, with the joint sustained activities of the folate and methionine metabolic cycles vital to ensure efficient metabolic disposal of homocysteine during pregnancy. These metabolic interdependencies between the two cycles highlight the need for adequate maternal intake of methionine, methyl donors such as choline (which undergoes oxidative metabolism to betaine; Barak and Tuma, 1983) and folate, as well as vitamin B_12_, vitamin B_6_ and vitamin B_2_ over pregnancy (Kalhan, 2016). Further, cord blood concentrations of folate, vitamin B_12_, vitamin B_6_ and, vitamin B_2_ are significantly higher than that in maternal blood (Kirshenbaum et al., 1987; Obeid et al., 2005), interpreted as there being active transport mechanisms for each of these nutrients across the placenta to meet fetal developmental demand (Solanky et al., 2010; Kalhan, 2016) and the high rates of homocysteine remethylation and transmethylation that occur as pregnancy advances (Kalhan and Marczewski, 2012). Precisely when along a developmental timeline these mechanisms are active and such maternal-fetal concentration gradients are generated is difficult to establish, but our previous data are consistent with the existence of active placental transport mechanisms for folate (Solanky et al., 2010) and amino acids (Mahendran et al., 1994) in the first trimester, with a positive correlation between maternal and placental methionine concentrations already present at this pregnancy stage (Jauniaux et al., 1998).

Hence, either sub-optimal maternal intakes of these nutrients or gene polymorphisms resulting in diminished catalytic activity of homocysteine metabolising enzymes, leading to allied perturbation of these metabolic cycles (Refsum et al., 1998; Selhub, 1999; Murphy and Fernandez-Ballart, 2011; Smith and Refsum, 2021), is associated with hyperhomocysteinemia (HHcy). HHcy is characterised by an elevation in the plasma concentration of total homocysteine (tHcy; > 15 µM), comprising the reduced and oxidized forms of homocysteine (Smith and Refsum, 2021). This has pleiotropic effects: raised maternal plasma concentrations of tHcy are associated with various vascular-related complications of pregnancy including pre-eclampsia, placental abruption, recurrent pregnancy loss, fetal growth restriction and stillbirth (Ray and Laskin, 1999; Vollset et al., 2000; Wang et al., 2000; de la Calle et al., 2003; Dodds et al., 2008; Murphy and Fernandez-Ballart, 2011; Pisal et al., 2019), as well as adverse outcomes for the baby such as NTDs and congenital abnormalities including heart defects (Vollset et al., 2000; de la Calle et al., 2003; Murphy and Fernandez-Ballart, 2011; Smith and Refsum, 2021) along with impaired gene methylation and regulation of gene activity (Fryer et al., 2009; Kim et al., 2009; Fryer et al., 2011).

### Homocysteine in Pregnancy–Maternal-Fetal Interrelationships

A few studies have performed longitudinal measurements to examine the changes in maternal tHcy concentration across each trimester of pregnancy, albeit in distinct populations with differing maternal diets and supplementary folic acid intakes (Cikot et al., 2001; Murphy et al., 2002; Murphy et al., 2004; Powers et al., 2004; Velzing-Aarts et al., 2005; Dodds et al., 2008). The relative expression and co-ordinated gestational activity of homocysteine-generating and homocysteine-metabolising enzymes will contribute as determinants of maternal tHcy concentration (Finkelstein, 1998, 2007). Pregnancy causes a lowering of plasma tHcy concentration compared to the non-pregnant state (Bonnette et al., 1998; Walker et al., 1999; Cikot et al., 2001; Murphy et al., 2002; Murphy et al., 2004; Powers et al., 2004) and although the pregnancy-related decline occurs independently of maternal folic acid supplementation use (Murphy et al., 2002; Murphy et al., 2004), the fall in tHcy concentration is greater with maternal folic acid supplementation (Murphy et al., 2002; Murphy et al., 2004). This physiological response to pregnancy has been suggested to be attributable to haemodilution and albumin binding of tHcy, with a correlation between maternal plasma albumin and tHcy concentrations reported previously (Walker et al., 1999). However, others do not hold with this view, showing that the degree of haemodilution and change in plasma albumin is far more marginal than can account for the marked pregnancy-related decline in tHcy concentration, and further, that this phenomenon is already evident prior to when maternal plasma volume begins to increase at ∼ 10 weeks gestation (Murphy et al., 2002). Others propose that the increased glomerular filtration rate and renal clearance of tHcy contributes, with a significant inverse correlation between tHcy plasma concentration and renal clearance of tHcy (Powers et al., 2004). Hence, in early first trimester there is a physiological shift to a lower maternal tHcy plasma concentration compared to the pre-pregnancy state (Murphy et al., 2002; Murphy et al., 2004; Powers et al., 2004), with mean maternal tHcy concentration declining further towards the second trimester (Murphy et al., 2002; Powers et al., 2004; Dodds et al., 2008) to a nadir at ∼20–28 weeks gestation (Andersson et al., 1992; Walker et al., 1999; Murphy et al., 2002; Velzing-Aarts et al., 2005), reaching a plateau by the third trimester (Murphy et al., 2002; Murphy et al., 2004; Powers et al., 2004; Velzing-Aarts et al., 2005), although some studies report an increase (Walker et al., 1999; López-Quesada et al., 2003), with a subsequent post-partum rise in maternal plasma tHcy concentration (Andersson et al., 1992; Cikot et al., 2001; Powers et al., 2004; Velzing-Aarts et al., 2005). As the pregnancy related decline in maternal tHcy concentration occurs independently of maternal folic acid supplementation (Murphy et al., 2002; Murphy et al., 2004) it has promoted the suggestion that this represents a physiological adaptation to pregnancy that may contribute to the regulation of haemostasis (Holmes, 2003), and which is most likely modulated by endocrine factors (Murphy et al., 2002), with estrogen a plausible regulatory candidate (Dimitrova et al., 2002; Holmes, 2003).

Understanding the mechanisms that influence and regulate maternal tHcy concentration is particularly important from a developmental perspective as the concentration of maternal tHcy at pre-conception and throughout human pregnancy is significantly correlated with fetal cord blood tHcy concentration measured at birth (Murphy et al., 2004), and as already alluded to, raised maternal tHcy concentration is associated with a variety of adverse pregnancy outcomes. Hence, maternal tHcy is a major determinant of fetal cord plasma tHcy concentration at birth, with maternal tHcy concentration higher than that in fetal plasma, with a significant, positive correlation between these two variables (Malinow et al., 1998; Wang et al., 2000; Molloy et al., 2002; Murphy et al., 2004; Pisal et al., 2019). This higher maternal concentration of tHcy relative to fetal concentration is likely to reflect homocysteine’s status as an amino acid which is not incorporated into protein. However, this contrasts with other non-proteinogenic amino acids such as taurine, where fetal taurine concentration is significantly higher than maternal (Cetin et al., 1992), reflective of its essential role in fetal growth and organogenesis as well as osmoregulation and neuromodulation (Sturman, 1988). Likewise, proteinogenic amino acids fetal amino acid concentrations are significantly higher than maternal (Cetin et al., 1992). Teleologically, this may protect the fetus from the potential teratogenic effects of homocysteine (van Mil et al., 2010), which are likely to be particularly impactful during the early stages of embryogenesis when organogenesis is occurring.

### Homocysteine—“Cross-Talk” Across the Maternal-Fetal Interface

The correlation between maternal and fetal tHcy concentrations, together with the biochemical similarity of homocysteine to methionine, led us to consider whether homocysteine could be actively transported across the epithelial exchange barrier of human placenta, the syncytiotrophoblast (Tsitsiou et al., 2009; Tsitsiou et al., 2011). Different classes of amino acid transporters are distributed to the maternal-facing microvillous plasma membrane and fetal-facing basal plasma membrane of the syncytiotrophoblast, providing amino acids for biosynthetic processes such as fetal protein synthesis as well as fetal metabolism (Cleal and Lewis, 2008).

Our focus was to examine the activity of the three amino acid transport systems that mediate methionine transport, namely systems L, A and y^+^L, and investigate whether homocysteine could act as an amino acid substrate for each of these amino acid transport mechanisms (Tsitsiou et al., 2009; Tsitsiou et al., 2011). We found that homoysteine could indeed be transported by each of these amino acid transporter mechanisms, in a manner similar to methionine (Tsitsiou et al., 2009). The contribution to placental homocysteine uptake was greatest for system L-mediated activity (69%), with lesser contributions by system A (21%) and system y^+^L-mediated (19%) activities (Tsitsiou et al., 2009). The dominance of system L-mediated activity is interesting, as this is a very rapid amino acid exchange mechanism that transports essential amino acids. It mediates placental uptake and fetal delivery of essential amino acids being distributed to both the microvillous and basal plasma membranes of the syncytiotrophoblast (Cleal and Lewis, 2008; Widdows et al., 2015). System L activity relies on the accumulative uptake of amino acids by system A to serve as substrates for exchange (Cleal and Lewis, 2008), and the ability of both systems to transport homocysteine presents a plausible route for transplacental homocysteine transfer to the developing fetus (Tsitsiou et al., 2011). The capacity of the placenta to take up and transport homocysteine could have several putative functional implications: reduction in the provision of amino acids to the developing fetus for growth and development by competing with other amino acid substrates for transport (Tsitsiou et al., 2009; Tsitsiou et al., 2011), induction of trophoblast cell apoptosis (Di Simone et al., 2003), altered vascular function (Wang et al., 2000; Laskowska et al., 2013), disturbed methylation capacity (Kim et al., 2009), altered placental development (Pickell et al., 2009) and perturbation of placental methionine/homocysteine metabolism (Kalhan, 2016). The latter could invoke a greater demand for placental folate availability as human placenta relies on the folate-dependent remethylation of homocysteine as the major pathway for its metabolic disposal, as BHMT expression is absent and CβS expression is minimal (Solanky et al., 2010) with undetectable or negligible CβS and cystathionase activities (Sturman et al., 1970; Gaull et al., 1972), which would serve to commit homocysteine to folate-dependent remethylation and the efficient recycling to methionine, thereby increasing the availability of methionine for protein synthesis and further cellular transmethylation reactions. For these reasons, this review focusses on this aspect of homocysteine metabolism.

There is a decreasing concentration gradient of plasma tHcy from maternal vein to umbilical vein to umbilical artery (Malinow et al., 1998). It has previously been reported that maternal plasma tHcy concentration is influenced by maternal folate and vitamin B_12_ status (Malinow et al., 1998; Refsum, 2001; Murphy et al., 2004), with an inverse relationship between these variables (Malinow et al., 1998; Molloy et al., 2002; Murphy et al., 2004; Yajnik et al., 2005; Gu et al., 2012). We (D’Souza et al., 2017) and others (Malinow et al., 1998) have shown that there is a positive correlation between umbilical venous and arterial tHcy concentration in cord blood at term in normal, uncomplicated pregnancies with a significantly higher concentration in umbilical venous plasma (D’Souza et al., 2017), indicating there is a small, but proportional uptake of homocysteine by the fetus, which could then be remethylated to methionine. A similar trend was seen as regards the umbilical venous-arterial concentration difference for vitamin B_12_, but not for folate, although study participants did report taking folic acid supplements in early pregnancy (D’Souza et al., 2017). Interestingly, cord venous tHcy exhibited a significant negative correlation with cord venous vitamin B_12_ concentration, but this was not observed for folate, suggesting tHcy entering the fetal circulation was predominantly modulated by vitamin B_12_ in this setting. The concept that fetal vitamin B_12_, in particular, exerts a regulatory dominance on fetal tHcy concentration is consistent with the observations of others (Monsen et al., 2001; Molloy et al., 2002; Molloy et al., 2005; Braekke et al., 2007), in agreement with our observations (D’Souza et al., 2017). Further support for this notion comes from the reciprocal relationship between cord venous tHcy and vitamin B_12_ concentrations with respect to anthropometric measures of neonates at birth: birth weight, corrected birth weight, length and mid-arm circumference were positively associated with cord venous tHcy, but negatively with cord venous vitamin B_12_ concentration, with no associations with cord venous folate (D’Souza et al., 2017). Interestingly, stratification of corrected birth weight percentiles showed a significant positive association with elevations in tHcy concentration in both cord venous and arterial plasma concentration, which, bearing in mind the aforementioned inverse relationship between tHcy and vitamin B_12_, may reflect an increased utilization of vitamin B_12_ in larger babies to remethylate homocysteine to methionine (D’Souza et al., 2017).

These relationships to fetal growth outcomes, led us to consider how the arterial concentration of tHcy might influence fetal vascular function, and in particular the distensibility of human placental chorionic plate arteries as a site of vascular resistance within the fetoplacental circulation (D’Souza et al., 2017). It is known that impairment of vasodilation is elicited by elevated tHcy concentrations in other vessel types (Tawakol et al., 1997; Chambers et al., 1999). We found a lower intrinsic stiffness and greater distensibility of placental chorionic vessels as the tHcy concentration in cord arterial plasma increased (D’Souza et al., 2017). A relatively greater distensibility of chorionic plate arteries would be consistent with a lower resistance in the fetoplacental circulation and the facilitation of placental nutrient delivery which would accord with the positive association between umbilical tHcy concentrations with birth weight outcomes taken as a measure of fetal growth.

Collectively, these observations suggest that within the setting of *normal* pregnancy, tHcy concentration in the maternal, placental and fetal compartments is highly regulated and is particularly modulated by maternal folate and vitamin B_12_ dietary intakes and is contingent upon the optimal synergistic activities of the folate and methionine metabolic cycles. The metabolic remethylation of homocysteine to methionine is responsible for the conversion of ∼50–80% of homocysteine generated (Bailey and Gregory, 1999), and as such represents a key functional locus at the intersection of homocysteine metabolism that will be particularly vulnerable to dysregulation associated with maternal folate and vitamin B_12_ dietary insufficiencies. This is likely to be particularly profound as regards fetal homocysteine metabolism, as BHMT activity is absent and the activity of transsulphuration pathway is negligible putting reliance on methyl-dependent transmethylation pathways to maintain homocysteine metabolic conversion to methionine, catalysed by methionine synthase (Figure 1). Crucially too, in the context of fetal growth and development, this remethylation conversion step is required to transfer a methyl group from 5-MTHF to homocysteine, thereby allowing tetrahydrofolate (THF) to be returned to the active folate pool for nucleotide biosynthesis (Bailey and Gregory, 1999; Figure 1). Hence, impaired methionine synthase activity results not only in the accumulation of homocysteine, but also 5-MTHF, leading to cellular depletion of other folate derivatives, a condition referred to as a “methyl trap”. The methyl trap arises because the reduction of 5,10-methyleneTHF to 5-MTHF by MTHFR (Figure 1) is irreversible, and methionine synthase is the only folate-dependent enzyme to utilise 5-MTHF as a substrate (Finkelstein, 1998; Bailey and Gregory, 1999; Beaudin and Stover, 2009). Hence, maintenance of catalytic activity at this metabolic locus requires both folate and vitamin B_12_ and is absolutely essential to promote normal embryonic/fetal development. This concept is further emphasized by the lack of embryonic viability that arises when the gene encoding methionine synthase is ablated in mice; homozygous-null embryos appear to survive through implantation but die shortly afterwards (Swanson et al., 2001).

### Homocysteine–a Metabolite With Potential to Act as a Signalling Molecule

The biochemical similarity of homocysteine to methionine means it can be recognized and activated by methionyl-tRNA synthetase (Jakubowski, 2001; Jakubowski, 2004; Jakubowski, 2006). However, homocysteine is not incorporated into protein as the error-editing activity of methionyl-tRNA synthetase produces homocysteine thiolactone (HTL) (Jakubowski, 2000; Jakubowski, 2003; Jakubowski, 2004; Perła-Kaja´n et al., 2007), which can be subsequently hydrolyzed by thiolactonases to homocysteine (Perła-Kaja´n et al., 2007), but which also interacts with free amino groups on the lysine residues of proteins leading to protein N-homocysteinylation (Jakubowski, 2003; Jakubowski, 2004; Jakubowski, 2006). Alternatively, homocysteine can form stable disulphide bonds with protein cysteine residues, leading to S-homocysteinylation, with both forms of homocysteinylation impairing protein functional activity (Perła-Kaja´et al., 2007). The degree of HTL synthesis will depend on the relative concentrations of homocysteine and methionine and the cellular metabolic capacities of methionine synthase and CβS as determinants of homocysteine concentration, together with the activity of methionyl-tRNA synthetase (Jakubowski, 1999). Because the activities of these two homocysteine metabolising enzymes requires folate, vitamin B_12_ and vitamin B_6_ (Figure 1), the rate of HTL formation and propensity for protein function impairment will depend on the concentration and availability of these micronutrients. For example, in human umbilical endothelial cells, deprivation of folate and vitamin B_12_ leads to homocysteine being incorporated into protein at a level which represents about ∼36% of that metabolized to methionine, which is attenuated to <0.3% when supplemented with folate and vitamin B_12_ (Jakubowski, 2000). Hence, protein homocysteinylation has the potential to impair embryonic/fetal development through impaired protein function (Jakubowski, 1999; Perła-Kaja´n et al., 2007), protein aggregation (Jakubowski, 1999), or the generation of autoantibodies directed against homocysteinylated proteins (Jakubowski, 2006; Perła-Kaja´n et al., 2007). Collectively, protein homocysteinylation can affect the function of both intracellular and extracellular proteins (Jakubowski, 2000) and so the function of a diverse array of proteins may be impacted, potentially affecting multiple biological processes.

However, it is worth commenting that elucidation of the molecular mechanisms by which HHcy elicits impaired physiological function leading to the pathogenesis of disease is difficult, as changes in the concentrations of individual homocysteine metabolites (homocysteine, SAH and HTL) are likely to occur in parallel making it difficult to attribute specific effects to an individual homocysteine metabolite *per se*. Notwithstanding this, as already outlined above, HHcy associates with an array of pregnancy complications, and in the broader context of disease pathogenesis there is a growing understanding that the homocysteine-methionine metabolic locus acts as a key metabolic sensor system. Imbalance of homocysteine-methionine cellular homeostasis is postulated to serve as a ‘metabolism-associated danger recognition signal’, through which modulation of SAM-dependent methylation and the hypomethylation of modified histone residues contributes to the epigenetic dysregulation and aetiology of a variety of metabolic, immune and vascular diseases (Shen et al., 2020).

Hence, perturbations in the metabolic interdependencies of homocysteine-methionine cycling are likely to have notable developmental impacts, particularly during the early phases of pregnancy when embryonic organogenesis is occurring with high rates of DNA synthesis and cellular proliferation together with rapid rates of *de novo* gene methylation (Geiman and Muegge, 2010). Indeed, the early phase of pregnancy represents a period of high embryonic developmental vulnerability that is critically dependent on the adequate provision of interacting nutrients involved in one-carbon metabolism, as most clearly exemplified in the case of NTDs, a group of congenital malformations of the central nervous system that arise from a failure of neural tube closure during embryogenesis. Sensitivity of neural tube closure to one-carbon metabolism is signified by the lowering of NTD risk associated with higher maternal folate (MRC Vitamin Study Research Group, 1991; Czeizel et al., 2011; De-Regil et al., 2015; Yadav et al., 2021), vitamin B_12_ (Ray et al., 2007; Gu et al., 2012; Molloy, 2018; Wahbeh and Manyama 2021; Yadav et al., 2021), vitamin B_6_ (Candito et al., 2008), choline (Shaw et al., 2004; Shaw et al., 2009), betaine (Shaw et al., 2004), and methionine (Shaw et al., 1997; Shaw et al., 2004) status.

These observations compel us to consider how these nutrients might reach the embryo from the mother over the period when the neural tube is closing to exert regulatory influence? This constitutes a developmentally important question, yet the mechanisms remain poorly defined. The embryonic neural plate undergoes a series of co-ordinated morphological events which culminates in formation of the closed neural tube by the fourth week post-conception (Greene and Copp, 2009; Greene and Copp, 2014). Failure of neural tube closure leads to NTDs, including anencephaly and spina bifida (Greene and Copp, 2009; Greene and Copp, 2014). Therefore, the temporal window over which nutrients have the potential to exert a modulatory influence on the orchestrated developmental processes that are involved in neural tube closure, as well as other embryonic organ systems in early pregnancy, necessitates that these nutrients reach the embryonic compartment over the timeframe of first few weeks of pregnancy (D’Souza et al., 2021). We would therefore propose that a better understanding of the pathways involved is needed if maternal dietary modulation of embryonic developmental events is being considered in the therapeutic context of clinical treatment.

With this in mind, in the next sections we focus on two aspects related to NTDs: 1) Based on the synergistic interaction of folate and vitamin B_12_ in maintaining methylation capacity (Figure 1), we consider whether methylation is a functional node at which folate and vitamin B_12_ converge to ameliorate NTD disease risk? 2) We discuss a putative transport pathway for the embryonic provision of folate and vitamin B_12_ based on their properties as homocysteine-lowering micronutrients which confer embryonic developmental benefit that lowers NTD risk, although knowledge remains relatively scant.

### Homocysteine, Methylation and Neural Tube Defect Risk

The folate-dependent amelioration of NTD risk, together with the knowledge that folate-dependent one-carbon metabolism generates SAM, the universal methyl donor for methylation (Figure 1), have led to a wealth of studies investigating whether these phenomena may be functionally linked, with altered methylation contributing to the aetiology of NTDs (Wilde et al., 2014). Although the molecular mechanisms by which methylation modulates neurulation and neural tube closure still remain to be fully defined, there is an emerging consensus that altered gene methylation and epigenetic regulation contribute to the complex aetiology of NTD risk (Blom et al., 2006; Blom, 2009; Greene et al., 2011; Imbard et al., 2013). Further, the functional activity of the methionine cycle is known to be essential to neural tube closure (Leung et al., 2017). Convincing evidence that methylation is a key locus to understanding the pathogenesis of NTDs comes from studies in cultured mouse embryos showing that the suppression of methylation cycle activity accompanied by a reduction in SAM and an increase in SAH concentration, likely to impair methyltransferase activity and methylation, was associated with the formation of cranial NTDs (Dunlevy et al., 2006b). Further studies have demonstrated that inhibition of methylation capacity or perturbation of homocysteine metabolism by methionine excess, resulting in a lowered SAM:SAH ratio, also results in the formation of cranial NTDs (Dunlevy et al., 2006a). Strikingly, these collective treatments were selective in impairing cranial neural tube closure, but did not result in other notable impacts as regards embryonic developmental growth, implicating a particular sensitivity of cranial neural tube closure to aberrant methylation. Consistent with this concept, *de novo* methylation has a key role in neural tube closure; ablation of the DNA methyltransferase 3b (*Dnmt3b*) gene gives rise to cranial NTDs in mice, yet this phenotype is not seen in *Dnmt3a* knockouts, which may relate to relatively high expression of *Dnmt3b* in the embryonic neural ectoderm and the elevating neural folds during neural tube formation (Okano et al., 1999). This may also relate to the capacity of *Dnmt3b* to methylate a broad subset of DNA target sites early in embryonic development as compared to *Dnmt3a*, including centromeric satellite repeats conferring chromatin stability (Okano et al., 1999).

Several studies, but not all, have found a significantly higher plasma tHcy concentration between mothers having a NTD-affected offspring compared to those with unaffected pregnancies (Tang et al., 2015; Yang et al., 2017). From their meta-analysis, Yang et al. (2017) concluded there was 6% (2–9%) higher tHcy concentration in mothers having a NTD-affected pregnancy compared to the control group, although they emphasize that the gestational timing of sampling with respect to the pregnancy-associated changes in maternal tHcy (as discussed above), maternal folate supplementation or mandatory folate fortification are likely to affect such associations.

Despite these observations, other evidence suggests that homocysteine *per se* is unlikely to have a direct causal role in the failure of neural tube closure. For example, NTDs are not seen in *Mthfr* ^−/−^ mice mutants that have a marked elevation in SAH or homocysteine accompanied by brain hypomethylation, and even with induced maternal folate deficiency, NTDs still do not arise in such mutants, yet offspring suffer from other developmental impacts such as restricted growth with developmental retardation and congenital heart defects (Chen et al., 2001; Li et al., 2005; Leung et al., 2017). Likewise, mouse mutants that lack CβS activity are born normal, without evidence of an increased incidence of NTDs, but have severe HHcy and exhibit marked growth restriction and die a few weeks after birth (Watanabe et al., 1995). But, perhaps the most compelling evidence for a lack of causality is that homocysteine (thiolactone) added exogenously to mouse embryo culture did not cause NTDs, although there was a dose-dependent increase in embryonic lethality and growth retardation (Greene et al., 2003). Likewise, others report a lack of NTD induction in mouse embryo cultures treated with HTL or a racemic mixture of D,L-homocysteine (Hansen et al., 2001) or in embryos where pregnant mice dams had been treated with D,L-homocysteine (Bennett et al., 2006). Together, these observations argue for a lack of a direct causative role of homocysteine in NTD pathogenesis but instead suggest that elevated homocysteine more likely serves a biomarker of dysregulated folate-dependent one-carbon metabolism that can impact on methylation events in a tissue-specific manner (Greene et al., 2003; Beaudin and Stover, 2009; Greene et al., 2011).

Supportive evidence that altered gene methylation is a downstream consequence of perturbed one-carbon metabolism which may link to NTD aetiology has come from studies that have examined methylation of long interspersed nucleotide element-1 (LINE-1) sequences; methylation signatures at these sequences are sensitive to nutritional factors such as methyl precursor availability in early embryonic development (Waterland and Jirtle, 2004). LINE-1 methylation is considered a good surrogate of genome wide DNA methylation, with significant correlations between these two variables demonstrated (Wang et al., 2010). In umbilical cord blood, LINE-1 methylation showed a significant inverse correlation with fetal plasma homocysteine concentration, but not folate (Fryer et al., 2009). Further analysis demonstrated that the majority of CpG dinucleotides located within CpG islands exhibited hypomethylation with the correlation to fetal plasma homocysteine concentration upheld (Fryer et al., 2011). Wang et al. (2010) demonstrated that methylation of genomic DNA (5-methyl cytosine) and LINE-1 sequences were lower in NTD-affected pregnancies with cephalic malformations compared to controls, with methylation levels of all CpG sites lower, but there was a lack of a significant correlation between either LINE-1 methylation levels or global methylation levels to maternal folate, vitamin B_12_, and tHcy concentrations across groups. However, in contrast, no differences were seen in the degree of methylation for spina bifida cases (Wang et al., 2010). In case-control studies examining fetal brain tissue, global DNA methylation levels were significantly decreased in NTD cases compared with controls, with DNA hypomethylation resulting in ∼5.7-fold increased risk for NTDs, whereas no methylation differences were seen in skin samples measured in parallel (Chen et al., 2010). This is in agreement with the observations of others that show a hypomethylation of genomic DNA in brain tissue of NTD cases compared to controls, which is not apparent in skin, lung, kidney or heart (Chang et al., 2011). DNA methylation profiling of the DNA repair gene O^6^ -methylguanine-DNA methyltransferase (*MGMT*) in DNA isolated from fetal brain tissue showed hypomethylation of the *MGMT* promoter was associated with an increased risk for cephalic malformations, especially among female embryos (Tran et al., 2012). However, others have failed to find aberrant genome-wide DNA methylation or CpG site-specific changes in the brain or spinal cord of anencephalic and spina bifida fetuses taken from a folate-replete population compared to pregnancies unaffected by NTDs as control, and whilst these tissues did not exhibit DNA methylation changes, interestingly, the fetal kidney exhibited multiple differentially methylated sites in the case of spina bifida (Price et al., 2016). An inherent difficulty in the experimental design of conducting these studies, which is not easily circumvented, is that the use of second trimester fetal tissues of NTD-affected pregnancies, if matched for gestational age to a NTD-unaffected control group, will, by necessity, have to include samples from other pregnancy complications, generating a heterogeneous reference control group. Notwithstanding this, such studies still provide mechanistic insights regarding the tissue-specific nature of NTD-associated methylation changes across different embryonic tissues.

In this context, others have examined placental tissue, as another fetal tissue of interest, revealing differential patterns of placental gene methylation between normal (at 40 weeks) and spina bifida (27–41 weeks) affected pregnancies, with the caveat that these were not gestation-matched. DNA methylation patterns for genes were differentially altered in the placentas of affected pregnancies but, interestingly, differential DNA methylation was most prominently altered in the transcription start site regions of genes functional in neurological system development, cell development and differentiation (Zhang et al., 2015). Yet, comparisons of chorionic villi DNA methylation between anencephaly to controls in other studies have yielded limited differences with only four CpG sites differentially methylated (Price et al., 2016).

However, whilst these collective observations implicate a role for methylation-dependent neural tube closure, this does not require the interdependent activities of the folate and methionine cycles and absent maternal or embryonic MTHFR activity does not impair neural tube closure, yet the functional activity of both cycles is crucial (Leung et al., 2017). Indeed, it has been suggested that under most conditions, the majority of one-carbon units for cytoplasmic processes are derived from mitochondrial formate, and consistent with this notion, ablation of the glycine cleavage system results in NTDs, with the function of the glycine cleavage system contributing to neural tube closure at all axial levels (Leung et al., 2017).

Whilst much emphasis has been placed on investigating DNA methylation, it should be remembered that altered methylation applies to other methylation acceptors such as proteins in the context of neural tube closure (Wilde et al., 2014). For example, adequate methylation of proteins affecting cellular cytoskeletal structure and motility such as actin and αβ-tubulin (Moephuli et al., 1997) and septin2 (Toriyama et al., 2017) have been shown to be required for neural tube closure. Recent attention has also been drawn to epigenetic regulation by histone methylation. In an initial small study of four cases of spina bifida and controls respectively using liquid chromatography with tandem mass spectrometry, the histone methylation pattern in fetal brain appeared altered in NTD cases; histone H3 lysine 79 dimethylation (H3K79me2) was detected in all controls, but was absent in NTD cases and, likewise, histone H2b lysine 5 monomethylation (H2bK5me) was also absent in NTD samples (Zhang et al., 2013). However, a more extensive analysis in 11 controls and 24 NTD cases by Western blotting demonstrated that expression of H3K79me2 was found to be very variable, with a reduced H3K37me2 expression in many NTD samples but not all (Zhang et al., 2013). This is interesting in the light of further studies that have demonstrated that homocysteinylation of histone H3 lysine 79 (H3K79Hcy) in neural stem cells is associated with the downregulation of genes related to neural tube closure, with several histone lysine residues identified as having the potential to be homocysteinylated (Zhang et al., 2018). Comparison of expression of H3K79Hcy in the fetal brains of NTD-affected cases with high homocysteine concentrations revealed a significantly higher level of H3K79Hcy compared to unaffected controls (Zhang et al., 2018). Hence, reduced histone methylation, or enhanced homocysteinylation, at specific histone lysine residues, can influence the transcriptional activity of genes implicated in neural tube closure.

Taken together, elevated homocysteine as a marker of perturbed folate-dependent methionine metabolism, together with accompanying impaired methylation appears to constitute a dysfunctional locus that is implicated in the aetiology of NTDs. Although multiple downstream methylation targets may be impacted by altered methylation, the early co-ordinated processes involved in neural tube closure seems particularly sensitive to such imbalances in methylation, with methylation regulation of DNA, histones and other proteins all likely to play a key role, in concert with other modes of epigenetic regulation (Greene et al., 2011; Wilde et al., 2014).

### Embryonic Provision of Folate and Vitamin B_12_

**FIGURE 2:**
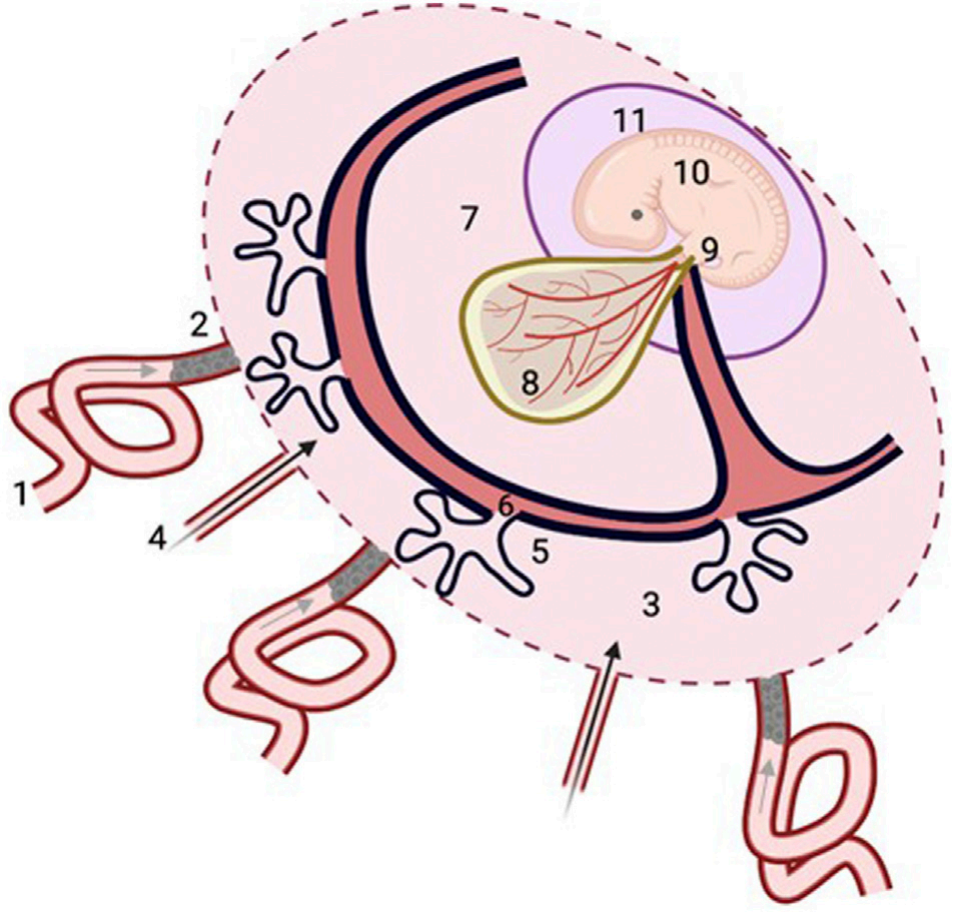
Histiotrophic pathway for embryonic nutrient delivery. Maternal plasma ultrafiltrates containing nutrients from the spiral arteries (1, grey arrow) percolate through plugs of endovascular trophoblast cells that occlude their openings (2), to reach the intervillous space of the placenta (3), along with secretions from the uterine glands (4, black arrow). Nutrients are taken up by the syncytiotrophoblast of the developing placenta (5), and pass through the villous mesenchymal core (6) with connections to the exocoelomic cavity (7) containing coelomic fluid with relatively high concentrations of methionine, folate and vitamin B_12_ (Table 1) which bathes the yolk sac (8). Nutrients taken up by the yolk sac from the coelomic fluid enter the vitelline circulation of the yolk sac (8) that is in direct continuity with the embryonic circulation, or else pass from the yolk sac cavity along the direct connection of the vitelline duct to the embryonic gut (9) of the embryo (10), which is contained within the amniotic cavity (11). Figure was created with BioRender.com.

As already outlined, neural tube closure in humans occurs by the 28th day post-conception (Greene and Copp, 2014). There is, however, concomitant development of the placenta over this period, and by the third week following conception, villi have already formed over the entire chorionic sac (Burton and Jauniaux, 2018a). Placental growth and development at this stage is believed to be supported by nutrient-containing secretions (“histiotroph”) from the underlying endometrial (uterine) glands, that flow directly through openings in the basal plate into the intervillous space surrounding the chorionic villi (Burton et al., 2002; Burton and Jauniaux, 2018a), as depicted in Figure 2. These secretions probably contain plasma transudates from the capillary plexus surrounding the glands as well as carbohydrates, lipids and proteins synthesised within the glandular cells, as well as other metabolites (Hempstock et al., 2004; Burton et al., 2007; Burton et al., 2011), together with a variety of growth factors likely to regulate placental development and differentiation (Burton et al., 2007; Burton et al., 2011; Burton and Jauniaux, 2018a). Whether these secretions contain micronutrients such as folate and vitamin B_12_ is not clear, but eminently plausible if maternal plasma transudates contribute to its composition. The glands gradually regress over the first trimester, but, until at least 10 weeks, remain connected with the placental intervillous space and their secretions appear to contribute to the fluid contained therein until this pregnancy stage (Burton et al., 2002; Hempstock et al., 2004).

So, over this phase of pregnancy, while the placenta is developing, neural tube closure is occurring concomitantly, yet, at this pregnancy stage, when embryonic demand for biosynthetic precursors is likely to be high to support the relatively rapid rates of embryonic growth (van Uitert et al., 2013), blood flow through the uteroplacental circulation has yet to be established. This is because prior to ∼10 weeks of gestation, maternal blood flow to the maternal spiral artery openings of the placenta is occluded by plugs of migrating endovascular trophoblast cells (Jaffe et al., 1997; Burton and Jauniaux, 2018a; Figure 2). Teleologically, this minimalisation of uteroplacental blood flow means that the embryo can undergo organogenesis and development in a low oxygen environment, limiting the damaging potential of reactive oxygen species (Burton et al., 2003), whilst also serving to promote placental development and differentiation (Burton and Jauniaux, 2018a). Hence, early embryonic and placental development occurs jointly in a low oxygen environment. Subsequently, at approximately 10 weeks of gestation, the enmeshed trophoblast cell “plugs” start to dislodge allowing the maternal arterial circulation to flow to the intervillous space, facilitating the full establishment of the uteroplacental circulation, alongside the contemporaneous elaboration of the feto-placental circulation (Burton and Jauniaux, 2018a; Burton and Jauniaux, 2018b), thereby enabling nutrients to be transferred from maternal blood flowing to the intervillous space, to fetal blood in the capillaries within the core of the placental villi *via* “haemotrophic” nutrition.

This poses the need to understand more fully the mechanistic pathways by which maternal nutrients are provided to the embryo when demand for nutrient precursors is going to be high, underscored by biosynthetic demand over the major phases of organogenesis and dynamic growth, and to elucidate the integrative metabolic networks that support such events whilst maintaining embryonic homeostasis to promote normal developmental progression. As already alluded to, the embryonic developmental landmark of neural tube closure appears to be notably “responsive” to maternal folate and vitamin B_12_ status (Imbard et al., 2013) which as described above, jointly participate in homocysteine metabolism and its remethylation to methionine (Figure 1), although it should be emphasized that the mechanistic loci at which these micronutrients act to promote neural tube closure may be independent and still remains unresolved. However, these phenomena in reducing NTD risk do prompt us to address a gap in knowledge and consider how these nutrients are delivered to the developing embryo?

This question is not easily answered as there is a scarcity of information. However, a more generic embryonic nutrient pathway of ‘histiotrophic nutrition’ has been described previously (Burton et al., 2001). In this scheme (Figure 2), the intercellular spaces between the endovascular trophoblast cells of the spiral artery “plugs” allows maternal plasma to permeate through the network of intercellular spaces between the enmeshed trophoblast cells and connect with the fluid within the intervillous space surrounding the chorionic (placental) villi (Burton et al., 1999). This fluid will therefore comprise a mixture of these plasma transudates and the aforementioned uterine gland secretions that transit directly to the intervillous space. Nutrients within the intervillous space fluid are then proposed to be taken up by the epithelial syncytiotrophoblast layer of the placental villi into the villous mesenchyme containing stromal channels that have connections to the exocoelomic cavity containing coelomic fluid, which surrounds and bathes the yolk sac for subsequent nutrient uptake by the yolk sac (Burton et al., 2001; Cindrova-Davies et al., 2017; Figure 2).

Nutrients taken up by the yolk sac can then pass to the embryo in direct continuity with the embryonic circulation *via* the vitelline circulation, or else can pass from the yolk sac cavity along the anatomical connection of the vitelline duct directly linked to the embryonic gut (D’Souza et al., 2021; Figure 2). Therefore, the human yolk sac is likely to play a crucial role in the provision of early embryonic nutrition (Burton et al., 2001; Cindrova-Davies et al., 2017) but knowledge regarding its nutrient transport function remains fairly scant. One reason for this is the limited gestational presence of the human yolk sac; it exists only to ∼12 weeks of human pregnancy after which it regresses (Jauniaux and Moscoso, 1992). It is worth commenting that the gestational timing of the regression of human yolk sac appears to be coincident with the onset and establishment of placental nutrient transport function, which, thereafter, will support nutrient demand for fetal growth and development.

Hence, over much of the first trimester, during critical periods of human embryonic development, transfer of nutrients and precursors for embryonic development and growth occurs prior to the onset of established blood flow through the placental circulations, with the human yolk sac likely to play a major role in the provision of embryonic nutrient delivery. In this context, we have recently proposed a role for the yolk sac in embryonic inositol transport that may contribute to the amelioration of risk for NTDs that are not responsive to folic acid (D’Souza et al., 2021).

In order for such a histiotrophic pathway scheme to support the human embryonic provision of folate and vitamin B_12_, it would require: 1) transporters for these micronutrients to be expressed and distributed to the first trimester human placental villi as well as the human yolk sac; and 2) favourable concentrations of these micronutrients in maternal plasma-derived transudates or uterine gland secretions that enter the intervillous space for uptake by transporters localised to placental villi, and in the coelomic fluid for yolk sac uptake for embryonic delivery.

In support of the first notion, the first trimester human placenta and yolk sac express transporting moieties in common for both micronutrients. We have previously described a scheme that describes the transplacental transport of folate in first trimester and term placenta involving folate receptor (FRα), proton-coupled folate transporter (PCFT) and reduced folate carrier (Solanky et al., 2010). By analogy, the co-expression of *FOLR1* and *SLC46A1* genes encoding FRα and PCFT respectively in human yolk sac (Cindrova-Davies et al., 2017), which are jointly required for FRα-mediated folate uptake and cellular release, leads to the speculation that this is a plausible mechanism, consistent with the localisation of FRα to the mesothelial surface of human yolk sac in contact with coelomic fluid (Jauniaux et al., 2007). A putative scheme to describe placental transcellular vitamin B_12_ transport, by analogy with other epithelia, is predicted to involve receptor-mediated binding of transcobalamin (TCN2) to its receptor (CD320), lysosomal export to the cytosol (LMBD1) and multidrug resistance protein 1 (MRP1)-mediated efflux (Nielsen et al., 2012). Expression of all four genes encoding these proteins is found in first trimester placenta and yolk sac transcriptomes (Cindrova-Davies et al., 2017), again leading to the possibility that this pathway of receptor-mediated endocytosis may be a mechanism for embryonic delivery of vitamin B_12_. Also supportive of this notion, is the distribution of megalin, an endocytic receptor for transcobalamin-B_12_, to the syncytiotrophoblast of first trimester placenta as well as the endoderm of human yolk sac (Burke et al., 2013).

In support of the second concept, Table 1 provides reported concentrations of precursors for one-carbon metabolism in maternal plasma and coelomic fluid and highlights the relatively high concentrations found in the latter, suggesting active transport mechanisms must be engaged at this stage of pregnancy to maintain such concentration gradients. It is also striking that there is a high relative concentration ratio of both methionine and vitamin B_12_ in coelomic fluid relative to maternal plasma, which is not as apparent for folate, yet homocysteine concentrations in coelomic fluid are maintained at a low level, in accord with the notion that this exerts the protective advantage of maintaining the embryo in an environment that will limit the potential for homocysteine-induced oxidative stress (Perna et al., 2003) and teratogenesis.

**TABLE 1:** Folate, vitamin B_12_ and methionine-related metabolites in maternal plasma and coelomic fluid.

Nutrient/metabolite	Maternal plasma	Coelomic fluid	References
Folate (nmol/L)	14.1	22.5	Campbell et al. (1993) ^§^
	13.2	9.0	Jauniaux et al. (2007)
Vitamin B_12_ (ng/L)	405	3680	Campbell et al. (1993)
Methionine (µmol/L)	11	46	Steegers-Theunissen et al. (1997)
	18	41	Jauniaux et al. (1994)
	21	48	Jauniaux et al. (1998)
Homocysteine (µmol/L)	8.7	2.5	Steegers-Theunissen et al. (1997)

^§^Given units (µg/L) converted for comparison.

Median/mean values over 6–12 weeks pregnancy.

Taken together, these findings are supportive of a postulate whereby the histiotrophic nutritional pathway, incorporating yolk sac-mediated uptake, plays a role in the embryonic provision of folate and vitamin B_12_.

## Conclusion

Optimal functioning of folate and methionine metabolic cycling, modulated by methionine, folate and vitamin B_12_ availability regulates homocysteine production. Perturbation of these metabolic cycles linked to one-carbon metabolism leads to the raised production of homocysteine and allied metabolites, which is associated with various adverse pregnancy outcomes including NTDs. The appropriate flux of one-carbon moieties through the folate and methionine metabolic cycles is essential for maintaining methylation capacity. The flux of one-carbon units through the methionine cycle, as well the partitioning of one-carbon donors from the mitochondrial glycine cleavage system to the folate cycle, is essential for neural tube closure necessitating the synergistic activities of both cycles (Leung et al., 2017). The flow and cycling of one-carbon metabolites relies on the sufficiency of nutrient cofactors to maintain effective folate and methionine metabolism, serving to direct one-carbon units and intermediates into the most favourable biosynthetic pathway to meet individual tissue and cellular requirement.

Given the intricate complexity and the multifactorial nature of human NTDs, delineation of methylated molecular targets and a more complete understanding of the regulatory mechanisms that maintain the metabolic interconnectivity that underpins methylation capacity, particularly in the embryonic period, remains an attractive area of research for developing future therapeutic approaches to reduce NTD risk. However, although a raised maternal provision of folate and vitamin B_12_ have been shown to be effective in the amelioration of NTD risk, the loci of their action remains to be determined. A more complete understanding of the route by which the transport of such nutrients from the mother to developing embryo is achieved, would be insightful in supporting the mechanistic framework that underpins nutrient-based therapeutics in the amelioration of embryonic developmental anomalies.

## Abbreviations

BHMT, betaine-homocysteine methyltransferase; CβS, cystathionine β-synthase; HHCy, hyperhomocysteinemia; 5-MTHF, 5-methyltetrahydrofolate; MTHFR, methylenetetrahydrofolate reductase; NTD, neural tube defect; SAM, S-adenosylmethionine; SAH, S-adenosylhomocysteine; tHcy, total homocysteine; THF, tetrahydofolate.

## References

[B1] Andersson A. Hultberg B. Brattström L. Isaksson A. (1992). Decreased Serum Homocysteine in Pregnancy. Eur. J. Clin. Chem. Clin. Biochem. 30, 377–379. 1511073

[B2] Bailey L. B. Gregory J. F. 3rd (1999). Folate Metabolism and Requirements. J. Nutr. 129, 779–782. 10.1093/jn/129.4.779 10203550

[B3] Barak A. J. Tuma D. J. (1983). Betaine, Metabolic By-Product or Vital Methylating Agent? Life Sci. 32, 771–774. 10.1016/0024-3205(83)90311-9 6338334

[B4] Beaudin A. E. Stover P. J. (2009). Insights into Metabolic Mechanisms Underlying Folate-Responsive Neural Tube Defects: a Minireview. Birth Defect Res. A 85, 274–284. 10.1002/bdra.20553 PMC4435943 19180567

[B5] Bennett G. D. Vanwaes J. Moser K. Chaudoin T. Starr L. Rosenquist T. H. (2006). Failure of Homocysteine to Induce Neural Tube Defects in a Mouse Model. Birth Defect Res. B 77, 89–94. 10.1002/bdrb.20071 16528705

[B6] Blom H. J. Shaw G. M. den Heijer M. Finnell R. H. (2006). Neural Tube Defects and Folate: Case Far from Closed. Nat. Rev. Neurosci. 7, 724–731. 10.1038/nrn1986 16924261 PMC2970514

[B7] Blom H. J. (2009). Folic Acid, Methylation and Neural Tube Closure in Humans. Birth Defect Res. A 85, 295–302. 10.1002/bdra.20581 19301298

[B8] Bonnette R. E. Caudill M. A. Boddie A. M. Hutson A. D. Kauwell G. P. A. Bailey L. B. (1998). Plasma homocyst(e)ine concentrations in pregnant and nonpregnant women with controlled folate intake. Obstetrics Gynecol. 92, 167–170. 10.1016/s0029-7844(98)00154-910.1097/00006250-199808000-00002 9699744

[B9] Bottiglieri T. (2002). S-Adenosyl-L-methionine (SAMe): from the Bench to the Bedside—Molecular Basis of a Pleiotrophic Molecule. Am. J. Clin. Nutr. 76 (Suppl. l), 1151S–7S. 10.1093/ajcn/76/5.1151S 12418493

[B10] Braekke K. Ueland P. M. Harsem N. K. Karlsen A. Blomhoff R. Staff A. C. (2007). Homocysteine, Cysteine, and Related Metabolites in Maternal and Fetal Plasma in Preeclampsia. Pediatr. Res. 62, 319–324. 10.1203/PDR.0b013e318123fba2 17622947

[B11] Burke K. A. Jauniaux E. Burton G. J. Cindrova-Davies T. (2013). Expression and Immunolocalisation of the Endocytic Receptors Megalin and Cubilin in the Human Yolk Sac and Placenta across Gestation. Placenta 34, 1105–1109. 10.1016/j.placenta.2013.08.003 23978537 PMC3820039

[B12] Burton G. J. Jauniaux E. (2018a). Pathophysiology of Placental-Derived Fetal Growth Restriction. Am. J. Obstet. Gynecol. 218 (2S), S745–S761. 10.1016/j.ajog.2017.11.577 29422210

[B13] Burton G. J. Jauniaux E. (2018b). Development of the Human Placenta and Fetal Heart: Synergic or Independent? Front. Physiol. 9, 373. 10.3389/fphys.2018.00373 29706899 PMC5906582

[B14] Burton G. J. Jauniaux E. Watson A. L. (1999). Maternal Arterial Connections to the Placental Intervillous Space during the First Trimester of Human Pregnancy: the Boyd Collection Revisited. Am. J. Obstetrics Gynecol. 181, 718–724. 10.1016/s0002-9378(99)70518-1 10486489

[B15] Burton G. J. Hempstock J. Jauniaux E. (2001). Nutrition of the Human Fetus during the First Trimester-A Review. Placenta 22 (22 Suppl. A), S70–S77. 10.1053/plac.2001.0639 11312634

[B16] Burton G. J. Watson A. L. Hempstock J. Skepper J. N. Jauniaux E. (2002). Uterine Glands Provide Histiotrophic Nutrition for the Human Fetus during the First Trimester of Pregnancy. J. Clin. Endocrinol. Metab. 87, 2954–2959. 10.1210/jcem.87.6.8563 12050279

[B17] Burton G. J. Hempstock J. Jauniaux E. (2003). Oxygen, Early Embryonic Metabolism and Free Radical-Mediated Embryopathies. Reprod. Biomed. Online 6, 84–96. 10.1016/s1472-6483(10)62060-3 12626148

[B18] Burton G. J. Jauniaux E. Charnock-Jones D. S. (2007). Human Early Placental Development: Potential Roles of the Endometrial Glands. Placenta 28 (28 Suppl. A), S64–S69. 10.1016/j.placenta.2007.01.007 17349689 PMC1878510

[B19] Burton G. J. Scioscia M. Rademacher T. W. (2011). Endometrial Secretions: Creating a Stimulatory Microenvironment within the Human Early Placenta and Implications for the Aetiopathogenesis of Preeclampsia. J. Reproductive Immunol. 89, 118–125. 10.1016/j.jri.2011.02.005 21531028

[B20] Campbell J. Wathen N. Perry G. Soneji S. Sourial N. Chard T. (1993). The Coelomic Cavity: an Important Site of Materno-Fetal Nutrient Exchange in the First Trimester of Pregnancy. Br. J. Obstet. Gynaecol. 100, 765–767. 10.1111/j.1471-0528.1993.tb14271.x 8399018

[B21] Candito M. Rivet R. Herbeth B. Boisson C. Rudigoz R.-C. Luton D. (2008). Nutritional and Genetic Determinants of Vitamin B and Homocysteine Metabolisms in Neural Tube Defects: a Multicenter Case-Control Study. Am. J. Med. Genet. 146A, 1128–1133. 10.1002/ajmg.a.32199 18386810

[B22] Cetin I. Marconi A. M. Corbetta C. Lanfranchi A. Baggiani A. M. Battaglia F. C. (1992). Fetal Amino Acids in Normal Pregnancies and in Pregnancies Complicated by Intrauterine Growth Retardation. Early Hum. Dev. 29, 183–186. 10.1016/0378-3782(92)90136-5 1396234

[B23] Chambers J. C. Obeid O. A. Kooner J. S. (1999). Physiological Increments in Plasma Homocysteine Induce Vascular Endothelial Dysfunction in Normal Human Subjects. Arterioscler. Thromb. Vasc. Biol. 19, 2922–2927. 10.1161/01.atv.19.12.2922 10591670

[B24] Chang H. Zhang T. Zhang Z. Bao R. Fu C. Wang Z. (2011). Tissue-specific Distribution of Aberrant DNA Methylation Associated with Maternal Low-Folate Status in Human Neural Tube Defects. J. Nutr. Biochem. 22, 1172–1177. 10.1016/j.jnutbio.2010.10.003 21333513

[B25] Chen Z. Karaplis A. C. Ackerman S. L. Pogribny I. P. Melnyk S. Lussier-Cacan S. (2001). Mice Deficient in Methylenetetrahydrofolate Reductase Exhibit Hyperhomocysteinemia and Decreased Methylation Capacity, with Neuropathology and Aortic Lipid Deposition. Hum. Mol. Genet. 10, 433–443. 10.1093/hmg/10.5.433 11181567

[B26] Chen X. Guo J. Lei Y. Zou J. Lu X. Bao Y. (2010). Global DNA Hypomethylation Is Associated with NTD-Affected Pregnancy: A Case-Control Study. Birth Defects Res. Part A Clin. Mol. Teratol. 88, 575–581. 10.1002/bdra.20670 20641100

[B27] Cikot R. J. L. M. Steegers-Theunissen R. P. M. Thomas C. M. G. de Boo T. M. Merkus H. M. W. M. Steegers E. A. P. (2001). Longitudinal Vitamin and Homocysteine Levels in Normal Pregnancy. Br. J. Nutr. 85, 49–58. 10.1079/bjn2000209 11227033

[B28] Cindrova-Davies T. Jauniaux E. Elliot M. G. Gong S. Burton G. J. Charnock-Jones D. S. (2017). RNA-seq Reveals Conservation of Function Among the Yolk Sacs of Human, Mouse, and Chicken. Proc. Natl. Acad. Sci. U. S. A. 114, E4753–E4761. 10.1073/pnas.1702560114 28559354 PMC5474779

[B29] Cleal J. K. Lewis R. M. (2008). The Mechanisms and Regulation of Placental Amino Acid Transport to the Human Foetus. J. Neuroendocrinol. 20, 419–426. 10.1111/j.1365-2826.2008.01662.x 18266945

[B30] Czeizel A. E. Dudás I. Paput L. Bánhidy F. (2011). Prevention of Neural-Tube Defects with Periconceptional Folic Acid, Methylfolate, or Multivitamins? Ann. Nutr. Metab. 58, 263–271. 10.1159/000330776 21865678

[B31] de la Calle M. Usandizaga R. Sancha M. Magdaleno F. Herranz A. Cabrillo E. (2003). Homocysteine, Folic Acid and B-Group Vitamins in Obstetrics and Gynaecology. Eur. J. Obstetrics Gynecol. Reproductive Biol. 107, 125–134. 10.1016/s0301-2115(02)00305-6 12648856

[B32] De-Regil L. M. Peña-Rosas J. P. Fernández-Gaxiola A. C. Rayco-Solon P. (2015). Effects and Safety of Periconceptional Oral Folate Supplementation for Preventing Birth Defects. Cochrane Database Syst. Rev. 12, CD007950. 10.1002/14651858.CD007950 PMC8783750 26662928

[B33] Di Simone N. Maggiano N. Caliandro D. Riccardi P. Evangelista A. Carducci B. (2003). Homocysteine Induces Trophoblast Cell Death with Apoptotic Features. Biol. Reproduction 69, 1129–1134. 10.1095/biolreprod.103.015800 12773435

[B34] Dimitrova K. DeGroot K. Myers A. K. Kim Y. D. (2002). Estrogen and Homocysteine. Cardiovasc. Res. 53, 577–588. 10.1016/s0008-6363(01)00462-x 11861028

[B35] Dodds L. Fell D. B. Dooley K. C. Armson B. A. Allen A. C. Nassar B. A. (2008). Effect of Homocysteine Concentration in Early Pregnancy on Gestational Hypertensive Disorders and Other Pregnancy Outcomes. Clin. Chem. 54, 326–334. 10.1373/clinchem.2007.097469 18070815

[B36] D'Souza S. W. Solanky N. Guarino J. Moat S. Sibley C. P. Taggart M. (2017). Human Placental Arterial Distensibility, Birth Weight, and Body Size Are Positively Related to Fetal Homocysteine Concentration. Reprod. Sci. 24, 1070–1078. 10.1177/1933719116678694 27879450

[B37] D'Souza S. W. Copp A. J. Greene N. D. E. Glazier J. D. (2021). Maternal Inositol Status and Neural Tube Defects: A Role for the Human Yolk Sac in Embryonic Inositol Delivery? Adv. Nutr. 12, 212–222. 10.1093/advances/nmaa100 32892218 PMC7849949

[B38] Ducker G. S. Rabinowitz J. D. (2017). One-carbon Metabolism in Health and Disease. Cell Metab. 25, 27–42. 10.1016/j.cmet.2016.08.009 27641100 PMC5353360

[B39] Dunlevy L. P. E. Burren K. A. Chitty L. S. Copp A. J. Greene N. D. E. (2006a). Excess Methionine Suppresses the Methylation Cycle and Inhibits Neural Tube Closure in Mouse Embryos. FEBS Lett. 580, 2803–2807. 10.1016/j.febslet.2006.04.020 16674949

[B40] Dunlevy L. P. E. Burren K. A. Mills K. Chitty L. S. Copp A. J. Greene N. D. E. (2006b). Integrity of the Methylation Cycle Is Essential for Mammalian Neural Tube Closure. Birth Defect Res. A 76, 544–552. 10.1002/bdra.20286 16933307

[B41] Finkelstein J. D. (1998). The Metabolism of Homocysteine: Pathways and Regulation. Eur. J. Pediatr. 157 (157 Suppl. l), S40–S44. 10.1007/pl00014300 9587024

[B42] Finkelstein J. D. (2007). Metabolic Regulatory Properties of S-Adenosylmethionine and S-Adenosylhomocysteine. Clin. Chem. Lab. Med. 45, 1694–1699. 10.1515/CCLM.2007.341 17963455

[B43] Fryer A. A. Nafee T. M. Ismail K. M. K. Carroll W. D. Emes R. D. Farrell W. E. (2009). LINE-1 DNA Methylation Is Inversely Correlated with Cord Plasma Homocysteine in Man: a Preliminary Study. Epigenetics 4, 394–398. 10.4161/epi.4.6.9766 19755846

[B44] Fryer A. A. Emes R. D. Ismail K. M. K. Haworth K. E. Mein C. Carroll W. D. (2011). Quantitative, High-Resolution Epigenetic Profiling of CpG Loci Identifies Associations with Cord Blood Plasma Homocysteine and Birth Weight in Humans. Epigenetics 6, 86–94. 10.4161/epi.6.1.13392 20864804 PMC3052917

[B45] Gaull G. Sturman J. A. Räihä N. C. R. (1972). Development of Mammalian Sulfur Metabolism: Absence of Cystathionase in Human Fetal Tissues. Pediatr. Res. 6, 538–547. 10.1203/00006450-197206000-00002 4625813

[B46] Geiman T. M. Muegge K. (2010). DNA Methylation in Early Development. Mol. Reprod. Dev. 77, 105–113. 10.1002/mrd.21118 19921744

[B47] Greene N. D. E. Copp A. J. (2009). Development of the Vertebrate Central Nervous System: Formation of the Neural Tube. Prenat. Diagn. 29, 303–311. 10.1002/pd.2206 19206138

[B48] Greene N. D. E. Copp A. J. (2014). Neural Tube Defects. Annu. Rev. Neurosci. 37, 221–242. 10.1146/annurev-neuro-062012-170354 25032496 PMC4486472

[B49] Greene N. D. Dunlevy L. P. Copp A. J. (2003). Homocysteine Is Embryotoxic but Does Not Cause Neural Tube Defects in Mouse Embryos. Anat. Embryol. 206, 185–191. 10.1007/s00429-002-0284-3 12592569

[B50] Greene N. D. E. Stanier P. Moore G. E. (2011). The Emerging Role of Epigenetic Mechanisms in the Etiology of Neural Tube Defects. Epigenetics 6, 875–883. 10.4161/epi.6.7.16400 21613818 PMC3154428

[B51] Gu Q. Li Y. Cui Z.-L. Luo X.-P. (2012). Homocysteine, Folate, Vitamin B12and B6in Mothers of Children with Neural Tube Defects in Xinjiang, China. Acta Paediatr. 101, e486–e490. 10.1111/j.1651-2227.2012.02795.x 22860981

[B52] Hansen D. K. Grafton T. F. Melnyk S. James S. J. (2001). Lack of Embryotoxicity of Homocysteine Thiolactone in Mouse Embryos *In Vitro* . Reprod. Toxicol. 15, 239–244. 10.1016/s0890-6238(01)00133-2 11390167

[B53] Hempstock J. Cindrova-Davies T. Jauniaux E. Burton G. J. (2004). Endometrial Glands as a Source of Nutrients, Growth Factors and Cytokines during the First Trimester of Human Pregnancy: a Morphological and Immunohistochemical Study. Reprod. Biol. Endocrinol. 2, 58. 10.1186/1477-7827-2-58 15265238 PMC493283

[B54] Holmes V. A. (2003). Changes in Haemostasis during Normal Pregnancy: Does Homocysteine Play a Role in Maintaining Homeostasis? Proc. Nutr. Soc. 62, 479–493. 10.1079/pns2003251 14506896

[B55] Imbard A. Benoist J.-F. Blom H. (2013). Neural Tube Defects, Folic Acid and Methylation. Int. J. Environ. Res. Public Health 10, 4352–4389. 10.3390/ijerph10094352 24048206 PMC3799525

[B56] Jaffe R. Jauniaux E. Hustin J. (1997). Maternal Circulation in the First-Trimester Human Placenta-Myth or Reality? Am. J. Obstetrics Gynecol. 176, 695–705. 10.1016/s0002-9378(97)70572-6 9077631

[B57] Jakubowski H. (1999). Protein Homocysteinylation: Possible Mechanism Underlying Pathological Consequences of Elevated Homocysteine Levels. FASEB J. 13, 2277–2283. 10.1096/fasebj.13.15.2277 10593875

[B58] Jakubowski H. Zhang L. Bardeguez A. Aviv A. (2000). Homocysteine Thiolactone: Metabolic Origin and Protein Homocysteinylation in Human. J. Nutr. 130 (2S Suppl. l), 377S–381S. 10.1093/jn/130.2.377S 10721911

[B59] Jakubowski H. (2001). Translational Accuracy of Aminoacyl-tRNA Synthetases: Implications for Atherosclerosis. J. Nutr. 131, 2983S–2987S. 10.1093/jn/131.11.2983S 11694633

[B60] Jakubowski H. (2003). Homocysteine-thiolactone and S-Nitroso-Homocysteine Mediate Incorporation of Homocysteine into Protein in Humans. Clin. Chem. Lab. Med. 41, 1462–1466. 10.1515/CCLM.2003.224 14656026

[B61] Jakubowski H. (2004). Molecular Basis of Homocysteine Toxicity in Humans. Cell. Mol. Life Sci. (CMLS) 61, 470–487. 10.1007/s00018-003-3204-7 14999406 PMC11138896

[B62] Jakubowski H. (2006). Pathophysiological Consequences of Homocysteine Excess. J. Nutr. 136 (6 Suppl. l), 1741S–1749S. 10.1093/jn/136.6.1741S 16702349

[B63] Jauniaux E. Moscoso J. (1992). “Morphology and Significance of the Human Yolk Sac,” in The First Twelve Weeks of Gestation. Editors Barnea E. R. Hustin J. Jauniaux E. (Berlin: Springer-Verlag), 192–213.

[B64] Jauniaux E. Sherwood R. A. Jurkovic D. Boa F. G. Campbell S. (1994). Pregnancy: Amino Acid Concentrations in Human Embryological Fluids. Hum. Reprod. 9, 1175–1179. 10.1093/oxfordjournals.humrep.a138654 7962397

[B65] Jauniaux E. Gulbis B. Gerlo E. Rodeck C. (1998). Free Amino Acid Distribution inside the First Trimester Human Gestational Sac. Early Hum. Dev. 51, 159–169. 10.1016/s0378-3782(97)00107-2 9605468

[B66] Jauniaux E. Johns J. Gulbis B. Spasic-Boskovic O. Burton G. J. (2007). Transfer of Folic Acid inside the First-Trimester Gestational Sac and the Effect of Maternal Smoking. Am. J. Obstet. Gynecol. 197, 58.e1. 10.1016/j.ajog.2007.02.009 17618758

[B67] Kalhan S. C. Marczewski S. E. (2012). Methionine, Homocysteine, One Carbon Metabolism and Fetal Growth. Rev. Endocr. Metab. Disord. 13, 109–119. 10.1007/s11154-012-9215-7 22418620

[B68] Kalhan S. C. (2016). One Carbon Metabolism in Pregnancy: Impact on Maternal, Fetal and Neonatal Health. Mol. Cell. Endocrinol. 435, 48–60. 10.1016/j.mce.2016.06.006 27267668 PMC5014566

[B69] Kim J.-M. Hong K. Lee J. H. Lee S. Chang N. (2009). Effect of Folate Deficiency on Placental DNA Methylation in Hyperhomocysteinemic Rats. J. Nutr. Biochem. 20, 172–176. 10.1016/j.jnutbio.2008.01.010 18547797

[B70] Kirshenbaum N. W. Dancis J. Levitz M. Lehanka J. Young B. K. (1987). Riboflavin Concentration in Maternal and Cord Blood in Human Pregnancy. Am. J. Obstetrics Gynecol. 157, 748–752. 10.1016/s0002-9378(87)80043-1 3631177

[B71] Laskowska M. Laskowska K. Terbosh M. Oleszczuk J. (2013). A Comparison of Maternal Serum Levels of Endothelial Nitric Oxide Synthase, Asymmetric Dimethylarginine, and Homocysteine in Normal and Preeclamptic Pregnancies. Med. Sci. Monit. 19, 430–437. 10.12659/MSM.883932 23739294 PMC3675838

[B72] Leung K.-Y. Pai Y. J. Chen Q. Santos C. Calvani E. Sudiwala S. (2017). Partitioning of One-Carbon Units in Folate and Methionine Metabolism Is Essential for Neural Tube Closure. Cell Rep. 21, 1795–1808. 10.1016/j.celrep.2017.10.072 29141214 PMC5699646

[B73] Li D. Pickell L. Liu Y. Wu Q. Cohn J. S. Rozen R. (2005). Maternal Methylenetetrahydrofolate Reductase Deficiency and Low Dietary Folate Lead to Adverse Reproductive Outcomes and Congenital Heart Defects in Mice. Am. J. Clin. Nutr. 82, 188–195. 10.1093/ajcn.82.1.18810.1093/ajcn/82.1.188 16002818

[B74] López-Quesada E. Vilaseca M.-A. Artuch R. Gómez E. Lailla J. M. (2003). Homocysteine and Other Plasma Amino Acids in Preeclampsia and in Pregnancies without Complications. Clin. Biochem. 36, 185–192. 10.1016/s0009-9120(03)00003-1 12726926

[B75] Mahendran D. Byrne S. Donnai P. D'Souza S. W. Glazier J. D. Jones C. J. P. (1994). Na^+^ Transport, H^+^ Concentration Gradient Dissipation, and System A Amino Acid Transporter Activity in Purified Microvillous Plasma Membrane Isolated from First-Trimester Human Placenta: Comparison with the Term Microvillous Membrane. Am. J. Obstetrics Gynecol. 171, 1534–1540. 10.1016/0002-9378(94)90397-2 7802063

[B76] Malinow M. R Rajkovic A. Barton Duell P. Hess D. L. Upson B. M. (1998). The relationship between maternal and neonatal umbilical cord plasma homocyst(e)ine suggests a potential role for maternal homocyst(e)ine in fetal metabolism. Am. J. Obstetrics Gynecol. 178, 228–233. 10.1016/s0002-9378(98)80005-7 9500479

[B77] Miyazawa H. Aulehla A. (2018). Revisiting the Role of Metabolism during Development. Development 145, dev131110. 10.1242/dev.131110 30275240

[B78] Moephuli S. R. Klein N. W. Baldwin M. T. Krider H. M. (1997). Effects of Methionine on the Cytoplasmic Distribution of Actin and Tubulin during Neural Tube Closure in Rat Embryos. Proc. Natl. Acad. Sci. U. S. A. 94, 543–548. 10.1073/pnas.94.2.543 9012820 PMC19549

[B79] Molloy A. M. Mills J. L. McPartlin J. Kirke P. N. Scott J. M. Daly S. (2002). Maternal and Fetal Plasma Homocysteine Concentrations at Birth: The Influence of Folate, Vitamin B12, and the 5,10-methylenetetrahydrofolate Reductase 677C-->T Variant. Am. J. Obstetrics Gynecol. 186, 499–503. 10.1067/mob.2002.121105 11904614

[B80] Molloy A. M. Mills J. L. Cox C. Daly S. F. Conley M. Brody L. C. (2005). Choline and Homocysteine Interrelations in Umbilical Cord and Maternal Plasma at Delivery. Am. J. Clin. Nutr. 82, 836–842. 10.1093/ajcn/82.4.836 16210714

[B81] Molloy A. M. (2018). Should Vitamin B12status Be Considered in Assessing Risk of Neural Tube Defects? Ann. N.Y. Acad. Sci. 1414, 109–125. 10.1111/nyas.13574 29377209 PMC5887889

[B82] Monsen A.-L. B. Ueland P. M. Vollset S. E. Guttormsen A. B. Markestad T. Solheim E. (2001). Determinants of Cobalamin Status in Newborns. Pediatrics 108, 624–630. 10.1542/peds.108.3.624 11533328

[B83] MRC Vitamin Study Research Group. (1991). Prevention of Neural Tube Defects: Results of the Medical Research Council Vitamin Study. Lancet 338 (8760), 131–137. 10.1016/0140-6736(91)90133-A 1677062

[B84] Murphy M. M. Fernandez-Ballart J. D. (2011). Homocysteine in Pregnancy. Adv. Clin. Chem. 53, 105–137. 10.1016/b978-0-12-385855-9.00005-9 21404916

[B85] Murphy M. M. Scott J. M. McPartlin J. M. Fernandez-Ballart J. D. (2002). The Pregnancy-Related Decrease in Fasting Plasma Homocysteine Is Not Explained by Folic Acid Supplementation, Hemodilution, or a Decrease in Albumin in a Longitudinal Study. Am. J. Clin. Nutr. 76, 614–619. 10.1093/ajcn/76.3.614 12198008

[B86] Murphy M. M. Scott J. M. Arija V. Molloy A. M. Fernandez-Ballart J. D. (2004). Maternal Homocysteine before Conception and throughout Pregnancy Predicts Fetal Homocysteine and Birth Weight. Clin. Chem. 50, 1406–1412. 10.1373/clinchem.2004.032904 15166109

[B87] Nielsen M. J. Rasmussen M. R. Andersen C. B. F. Nexø E. Moestrup S. K. (2012). Vitamin B12 Transport from Food to the Body's Cells-A Sophisticated, Multistep Pathway. Nat. Rev. Gastroenterol. Hepatol. 9, 345–354. 10.1038/nrgastro.2012.76 22547309

[B88] Obeid R. Munz W. Jäger M. Schmidt W. Herrmann W. (2005). Biochemical Indexes of the B Vitamins in Cord Serum Are Predicted by Maternal B Vitamin Status. Am. J. Clin. Nutr. 82, 133–139. 10.1093/ajcn.82.1.13310.1093/ajcn/82.1.133 16002811

[B89] Okano M. Bell D. W. Haber D. A. Li E. (1999). DNA Methyltransferases Dnmt3a and Dnmt3b Are Essential for De Novo Methylation and Mammalian Development. Cell 99, 247–257. 10.1016/s0092-8674(00)81656-6 10555141

[B90] Perła-Kaján J. Twardowski T. Jakubowski H. (2007). Mechanisms of Homocysteine Toxicity in Humans. Amino Acids 32, 561–572. 10.1007/s00726-006-0432-9 17285228

[B91] Perna A. F. Ingrosso D. De Santo N. G. (2003). Homocysteine and Oxidative Stress. Amino Acids 25, 409–417. 10.1007/s00726-003-0026-8 14661100

[B92] Pickell L. Li D. Brown K. Mikael L. G. Wang X.-L. Wu Q. (2009). Methylenetetrahydrofolate Reductase Deficiency and Low Dietary Folate Increase Embryonic Delay and Placental Abnormalities in Mice. Birth Defect Res. A 85, 531–541. 10.1002/bdra.20575 19215022

[B93] Pisal H. Dangat K. Randhir K. Khaire A. Mehendale S. Joshi S. (2019). Higher Maternal Plasma Folate, Vitamin B12 and Homocysteine Levels in Women with Preeclampsia. J. Hum. Hypertens. 33, 393–399. 10.1038/s41371-019-0164-4 30647465

[B94] Powers R. W. Majors A. K. Kerchner L. J. Conrad K. P. (2004). Renal Handling of Homocysteine during Normal Pregnancy and Preeclampsia. J. Soc. Gynecol. Investigation 11, 45–50. 10.1016/j.jsgi.2003.07.007 14706683

[B95] Price E. M. Peñaherrera M. S. Portales-Casamar E. Pavlidis P. Van Allen M. I. McFadden D. E. (2016). Profiling Placental and Fetal DNA Methylation in Human Neural Tube Defects. Epigenetics Chromatin 9, 6. 10.1186/s13072-016-0054-8 26889207 PMC4756451

[B96] Ray J. G. Laskin C. A. (1999). Folic acid and homocyst(e)ine metabolic defects and the risk of placental abruption, pre-eclampsia and spontaneous pregnancy loss: A systematic review. Placenta 20, 519–529. 10.1053/plac.1999.0417 10452905

[B97] Ray J. G. Wyatt P. R. Thompson M. D. Vermeulen M. J. Meier C. Wong P.-Y. (2007). Vitamin B12 and the Risk of Neural Tube Defects in a Folic-Acid-Fortified Population. Epidemiology 18, 362–366. 10.1097/01.ede.0000257063.77411.e9 17474166

[B98] Refsum H. Ueland P. M. Nygård O. Vollset S. E. (1998). Homocysteine and Cardiovascular Disease. Annu. Rev. Med. 49, 31–62. 10.1146/annurev.med.49.1.31 9509248

[B99] Refsum H. (2001). Folate, Vitamin B12 and Homocysteine in Relation to Birth Defects and Pregnancy Outcome. Br. J. Nutr. 85 (Suppl. l), S109–S113. 10.1049/BJN200030210.1079/bjn2000302 11509098

[B100] Selhub J. (1999). Homocysteine Metabolism. Annu. Rev. Nutr. 19, 217–246. 10.1146/annurev.nutr.19.1.217 10448523

[B101] Shaw G. M. Velie E. M. Schaffer D. M. (1997). Is Dietary Intake of Methionine Associated with a Reduction in Risk for Neural Tube Defect-Affected Pregnancies? Teratology 56, 295–299. 10.1002/(sici)1096-9926(199711)56:5<295::aid-tera1>3.0.co;2-x 9451752

[B102] Shaw G. M. Carmichael S. L. Yang W. Selvin S. Schaffer D. M. (2004). Periconceptional Dietary Intake of Choline and Betaine and Neural Tube Defects in Offspring. Am. J. Epidemiol. 160, 102–109. 10.1093/aje/kwh187 15234930

[B103] Shaw G. M. Finnell R. H. Blom H. J. Carmichael S. L. Vollset S. E. Yang W. (2009). Choline and Risk of Neural Tube Defects in a Folate-Fortified Population. Epidemiology 20, 714–719. 10.1097/EDE.0b013e3181ac9fe7 19593156

[B104] Shen W. Gao C. Cueto R. Liu L. Fu H. Shao Y. (2020). Homocysteine-methionine Cycle Is a Metabolic Sensor System Controlling Methylation-Regulated Pathological Signaling. Redox Biol. 28, 101322. 10.1016/j.redox.2019.101322 31605963 PMC6812029

[B105] Smith A. D. Refsum H. (2021). Homocysteine - from Disease Biomarker to Disease Prevention. J. Intern. Med. 290, 826–854. 10.1111/joim.13279 33660358

[B106] Solanky N. Requena Jimenez A. D'Souza S. W. Sibley C. P. Glazier J. D. (2010). Expression of Folate Transporters in Human Placenta and Implications for Homocysteine Metabolism. Placenta 31, 134–143. 10.1016/j.placenta.2009.11.017 20036773

[B107] Steegers-Theunissen R. P. M. Wathen N. C. Eskes T. K. A. B. Raaij-Selten B. Chard T. (1997). Maternal and Fetal Levels of Methionine and Homocysteine in Early Human Pregnancy. Br. J. Obstet. Gynaecol. 104, 20–24. 10.1111/j.1471-0528.1997.tb10642.x 8988690

[B108] Strain J. J. Dowey L. Ward M. Pentieva K. McNulty H. (2004). B-vitamins, Homocysteine Metabolism and CVD. Proc. Nutr. Soc. 63, 597–603. 10.1079/pns2004390 15831132

[B109] Sturman J. A. Rassin D. K. Gaull G. E. (1970). Distribution of Transsulphuration Enzymes in Various Organs and Species. Int. J. Biochem. 1, 251–253. 10.1016/0020-711X(70)90102-3

[B110] Sturman J. A. (1988). Taurine in Development. J. Nutr. 118, 1169–1176. 10.1093/jn/118.10.1169 3054019

[B111] Swanson D. A. Liu M.-L. Baker P. J. Garrett L. Stitzel M. Wu J. (2001). Targeted Disruption of the Methionine Synthase Gene in Mice. Mol. Cell. Biol. 21, 1058–1065. 10.1128/MCB.21.4.1058-1065.2001 11158293 PMC99560

[B112] Tang K.-F. Li Y.-L. Wang H.-Y. (2015). Quantitative Assessment of Maternal Biomarkers Related to One-Carbon Metabolism and Neural Tube Defects. Sci. Rep. 5, 8510. 10.1038/srep08510 25728980 PMC4345334

[B113] Tawakol A. Omland T. Gerhard M. Wu J. T. Creager M. A. (1997). Hyperhomocyst(e)inemia is associated with impaired endothelium-dependent vasodilation in humans. Circulation 95, 1119–1121. 10.1161/01.cir.95.5.1119 9054838

[B114] Toriyama M. Toriyama M. Wallingford J. B. Finnell R. H. (2017). Folate‐dependent Methylation of Septins Governs Ciliogenesis during Neural Tube Closure. FASEB J. 31, 3622–3635. 10.1096/fj.201700092R 28432198 PMC5503710

[B115] Tran S. Wang L. Le J. Guan J. Wu L. Zou J. (2012). Altered Methylation of the DNA Repair Gene MGMT Is Associated with Neural Tube Defects. J. Mol. Neurosci. 47, 42–51. 10.1007/s12031-011-9676-2 22101741

[B116] Tsitsiou E. Sibley C. P. D'Souza S. W. Catanescu O. Jacobsen D. W. Glazier J. D. (2009). Homocysteine Transport by Systems L, A and y+L across the Microvillous Plasma Membrane of Human Placenta. J. Physiol. 587, 4001–4013. 10.1113/jphysiol.2009.173393 19564394 PMC2756434

[B117] Tsitsiou E. Sibley C. P. D’Souza S. W. Catanescu O. Jacobsen D. W. Glazier J. D. (2011). Homocysteine Is Transported by the Microvillous Plasma Membrane of Human Placenta. J. Inherit. Metab. Dis. 34, 57–65. 10.1007/s10545-010-9141-3 20567909 PMC2966547

[B118] van Mil N. H. Oosterbaan A. M. Steegers-Theunissen R. P. M. (2010). Teratogenicity and Underlying Mechanisms of Homocysteine in Animal Models: a Review. Reprod. Toxicol. 30, 520–531. 10.1016/j.reprotox.2010.07.002 20656016

[B119] van Uitert E. M. Exalto N. Burton G. J. Willemsen S. P. Koning A. H. J. Eilers P. H. C. (2013). Human Embryonic Growth Trajectories and Associations with Fetal Growth and Birthweight. Hum. Reprod. 28, 1753–1761. 10.1093/humrep/det115 23569080

[B120] Velzing-Aarts F. V. Holm P. I. Fokkema M. R. van der Dijs F. P. Ueland P. M. Muskiet F. A. (2005). Plasma Choline and Betaine and Their Relation to Plasma Homocysteine in Normal Pregnancy. Am. J. Clin. Nutr. 81, 1383–1389. 10.1093/ajcn/81.6.1383 15941891

[B121] Vollset S. E. Refsum H. Irgens L. M. Emblem B. M. Tverdal A. Gjessing H. K. (2000). Plasma Total Homocysteine, Pregnancy Complications, and Adverse Pregnancy Outcomes: the Hordaland Homocysteine Study. Am. J. Clin. Nutr. 71, 962–968. 10.1093/ajcn/71.4.962 10731504

[B122] Wahbeh F. Manyama M. (2021). The Role of Vitamin B12 and Genetic Risk Factors in the Etiology of Neural Tube Defects: A Systematic Review. Int. J. Dev. Neurosci. 81, 386–406. 10.1002/jdn.10113 33851436

[B123] Walker M. C. Smith G. N. Perkins S. L. Keely E. J. Garner P. R. (1999). Changes in Homocysteine Levels during Normal Pregnancy. Am. J. Obstetrics Gynecol. 180, 660–664. 10.1016/s0002-9378(99)70269-3 10076144

[B124] Wang J. Trudinger B. J. Duarte N. Wilcken D. E. Li Wang X. (2000). Elevated circulating homocyst(e)ine levels in placental vascular disease and associated pre-eclampsia. BJOG 107, 935–938. 10.1111/j.1471-0528.2000.tb11095.x 10901568

[B125] Wang L. Wang F. Guan J. Le J. Wu L. Zou J. (2010). Relation between Hypomethylation of Long Interspersed Nucleotide Elements and Risk of Neural Tube Defects. Am. J. Clin. Nutr. 91, 1359–1367. 10.3945/ajcn.2009.28858 20164316

[B126] Watanabe M. Osada J. Aratani Y. Kluckman K. Reddick R. Malinow M. R. (1995). Mice deficient in cystathionine beta-synthase: animal models for mild and severe homocyst(e)inemia. Proc. Natl. Acad. Sci. U.S.A. 92, 1585–1589. 10.1073/pnas.92.5.1585 7878023 PMC42564

[B127] Waterland R. A. Jirtle R. L. (2004). Early Nutrition, Epigenetic Changes at Transposons and Imprinted Genes, and Enhanced Susceptibility to Adult Chronic Diseases. Nutrition 20, 63–68. 10.1016/j.nut.2003.09.011 14698016

[B128] Widdows K. L. Panitchob N. Crocker I. P. Please C. P. Hanson M. A. Sibley C. P. (2015). Integration of Computational Modeling with Membrane Transport Studies Reveals New Insights into Amino Acid Exchange Transport Mechanisms. FASEB J. 29, 2583–2594. 10.1096/fj.14-267773 25761365 PMC4469330

[B129] Wilde J. J. Petersen J. R. Niswander L. (2014). Genetic, Epigenetic, and Environmental Contributions to Neural Tube Closure. Annu. Rev. Genet. 48, 583–611. 10.1146/annurev-genet-120213-092208 25292356 PMC4649936

[B130] Williamson C. S. (2016). Nutrition in Pregnancy. Nutr. Bull. 31, 28–59. 10.1111/j.1467-3010.2006.00541.x

[B131] Yadav U. Kumar P. Rai V. (2021). Maternal Biomarkers for Early Prediction of the Neural Tube Defects Pregnancies. Birth Defects Res. 113, 589–600. 10.1002/bdr2.1842 33188559

[B132] Yajnik C. S. Deshpande S. S. Panchanadikar A. V. Naik S. S. Deshpande J. A. Coyaji K. J. (2005). Maternal Total Homocysteine Concentration and Neonatal Size in India. Asia Pac. J. Clin. Nutr. 14, 179–181. 15927937

[B133] Yang M. Li W. Wan Z. Du Y. (2017). Elevated Homocysteine Levels in Mothers with Neural Tube Defects: a Systematic Review and Meta-Analysis. J. Maternal-Fetal Neonatal Med. 30, 2051–2057. 10.1080/14767058.2016.1236248 27633659

[B134] Zhang Q. Xue P. Li H. Bao Y. Wu L. Chang S. (2013). Histone Modification Mapping in Human Brain Reveals Aberrant Expression of Histone H3 Lysine 79 Dimethylation in Neural Tube Defects. Neurobiol. Dis. 54, 404–413. 10.1016/j.nbd.2013.01.014 23376398

[B135] Zhang X. Pei L. Li R. Zhang W. Yang H. Li Y. (2015). Spina Bifida in Fetus Is Associated with an Altered Pattern of DNA Methylation in Placenta. J. Hum. Genet. 60, 605–611. 10.1038/jhg.2015.80 26178427

[B136] Zhang Q. Bai B. Mei X. Wan C. Cao H. Dan Li D. (2018). Elevated H3K79 Homocysteinylation Causes Abnormal Gene Expression during Neural Development and Subsequent Neural Tube Defects. Nat. Commun. 9, 3436. 10.1038/s41467-018-05451-7 30143612 PMC6109101

